# Using machine learning for predicting cervical cancer from Swedish electronic health records by mining hierarchical representations

**DOI:** 10.1371/journal.pone.0237911

**Published:** 2020-08-21

**Authors:** Rebecka Weegar, Karin Sundström

**Affiliations:** 1 Department of Computer and Systems Sciences, Stockholm University, Kista, Sweden; 2 Department of Laboratory Medicine, Karolinska Institutet, Stockhom, Sweden; University of Oxford, UNITED KINGDOM

## Abstract

Electronic health records (EHRs) contain rich documentation regarding disease symptoms and progression, but EHR data is challenging to use for diagnosis prediction due to its high dimensionality, relative scarcity, and substantial level of noise. We investigated how to best represent EHR data for predicting cervical cancer, a serious disease where early detection is beneficial for the outcome of treatment. A case group of 1321 patients with cervical cancer were matched to ten times as many controls, and for both groups several types of events were extracted from their EHRs. These events included clinical codes, lab results, and contents of free text notes retrieved using a LSTM neural network. Clinical events are described with great variation in EHR texts, leading to a very large feature space. Therefore, an event hierarchy inferred from the textual events was created to represent the clinical texts. Overall, the events extracted from free text notes contributed the most to the final prediction, and the hierarchy of textual events further improved performance. Four classifiers were evaluated for predicting a future cancer diagnosis where Random Forest achieved the best results with an AUC of 0.70 from a year before diagnosis up to 0.97 one day before diagnosis. We conclude that our approach is sound and had excellent discrimination at diagnosis, but only modest discrimination capacity before this point. Since our study objective was earlier disease prediction than such, we propose further work should consider extending patient histories through e.g. the integration of primary health records preceding referral to hospital.

## Introduction

Information on disease progression documented in electronic health records (EHRs) is a potential source of valuable new knowledge which could lead to improved health care [1–3]. Since EHR information is derived directly from health care, there is a great interest on how to best use this source for real-life applications by way of advanced medical informatics. Applications that can benefit from EHR mining include clinical decision support, adverse event detection, and risk prediction [4]. One important potential of mining of EHRs is to generate new clinical hypotheses, and since EHRs document large populations observed over time, they allow for investigations regarding the relationship between clinical events and outcomes [4].

Compared to cohort studies, where specific information about individuals is typically collected at predefined time intervals, the use of EHR data has several distinct strengths but also limitations. Some of the advantages of using EHR data are that data are collected continuously, that a richer set of information types is included, and that less resources may be required to acquire the data. However, since EHRs are not primarily used to collect data for research purposes, EHR data are typically sparse and can contain a high level of noise, making this type of data challenging to analyze [5].

In this work, the focus is prediction of a major human cancer, i.e. cervical cancer, using EHRs as input. Globally, cervical cancer is one of the dominating cancer forms in women, with half a million cases each year. In Sweden, due to prevention through organized cervical screening, the disease is rarer but there are still about 550 cases of cervical cancer per year and the incidence has risen in the past two years. The median age of women getting the diagnosis is 46 years and thus it is a disease which strikes relatively early in life [6]. Improved prediction of risk could lead to interventions at an earlier stage, where the illness may still exist as a precancerous lesions amenable to surgical removal. For cancer diseases, earlier diagnosis could also lead to improvements in the outcomes of treatment and quality of life as well as for survival [7], and for cervical cancer relative survival is higher when the cancer is detected at an early stage [6]. The latter improvement is termed down-staging, and would be of value especially in a disease such as cervical cancer, where higher stages are associated with very high mortality. Machine learning models created from EHRs could, if sufficiently accurate, potentially lead to an earlier diagnosis and increased knowledge regarding the events preceding a diagnosis. In this work, the aim is therefore to apply machine learning to EHRs and to explore how well classifiers can identify future cervical cancer cases. To this end, both the informativeness of different event types found in EHRs (diagnosis codes, drug codes, free text, procedures, and lab results) was evaluated together with the issue of how to best represent such events for diagnosis prediction.

Machine learning methods have been applied to EHRs for predicting a number of different outcomes, both for specific diseases and also for the risk of mortality and hospitalization, and the number of studies using EHR data for creating risk prediction models is increasing [5].

Zhao and Weng [8] used 20 variables known to be related to pancreatic cancer and weighted them using PubMed abstracts. The variables included symptoms, lab-results and comorbidities, and were extracted from EHRs for 98 cases and 196 controls. Each selected variable was assigned a weight according to if the association to pancreatic cancer mined from the abstracts was positive or negative and a Bayesian Network Inference model using the assigned weights gave a better predictive performance compared to not using the weights derived from PubMed.

In their study on using machine learning to develop risk prediction models from health record data Mani et al. [9] aimed to identify patients at risk of type 2 diabetes. They extracted 16 variables representing demographic information, clinical findings and laboratory values for over 2,000 patients, where of 10% were patients with a diabetes type 2 diagnosis and the remaining were controls. Three data sets representing different time intervals were created. In the first one, all data up to the diagnosis date were included, while in the second and third ones, data were included from over 365 days and over 180 days before the diagnosis. A number of classifiers were evaluated for predicting which patients would develop type 2 diabetes, and an AUC of >0.8 was achieved for each of the three time intervals.

Huang et al. [10] aimed to predict future cases of depression, the severity of the depression, and the response to treatment. In brief, 5,000 patients with a future depression diagnosis and at least 1.5 years of data before the diagnosis were matched to six times as many controls. Diagnosis codes, medication codes, demographics and free text from EHRs belonging to the cases and controls were used as input and their model could predict depression diagnoses 12 months in advance with an AUC (area under the curve) of 0.7, and when including all available data up to the diagnosis data, the corresponding result was an AUC of 0.8.

Kop et al. [11] used structured data from EHRs to predict future cases of colorectal cancer (CRC). Six months of data for over 260,000 patients, whereof 0.5% CRC cases, were extracted from primary care health records. The data included ICPC codes (International Classification of Primary Care), ATC codes (Anatomical Therapeutic Chemical Classification System) and laboratory values. Temporal and co-occurrence patterns were mined from the data set and used as input for three different classifiers, CART decision trees, logistic regression and Random Forest. An AUC of 0.891 was achieved and the input features were ranked according to the importance factor provided by the logistic regression model. Most of the features with high ranks corresponded to events known to be linked to colorectal cancer, providing validation of their model.

Further promising examples of studies have used neural network models such denoising autoencoders in combination with Random Forest, convolutional networks, and recurrent networks for risk prediction of subsequent clinically relevant diseases through the use of EPR data [12–14].

## Materials and methods

### Ethics statement

The use of health record data in this project was approved by the Regional Ethical Review Board in Stockholm, Sweden, which determined that informed consent from the study participants was not required. The data was anonymized before being accessed by the researchers. Ethical permission number: 2014/1882-31/5.

### Feature extraction

When health records are mined, a first necessary step is feature extraction, during which, data of interest are selected and extracted from the records. There are two possible ways of selecting which features to include, either a top-down approach is used, where domain or expert knowledge guides the feature selection, or a bottom-up approach is applied, with an open, data driven feature selection [1]. A benefit of using the bottom up approach is that it allows for detecting new, previously unknown links between events and outcomes, since no a priori assumptions are made regarding which features are relevant to include in the model [11].

EHRs contain several information types requiring different levels of pre-processing before they can be included in a machine learning model. Often, a distinction is made between structured and unstructured information, where diagnosis codes, drug codes, and demographic information such as age or gender is considered as structured, and therefore easier to represent for machine learning purposes. Free text, on the other hand, is regarded as unstructured information as it is not possible to directly determine the value or meaning of an EHR free text note. This free text makes up a substantial part of the documentation in EHRs, and since it describes a patient’s health status, symptoms, and treatments, it is potentially valuable to include free text in risk prediction models. Free text notes require a higher level of pre-processing and therefore Natural Language Processing methods may be applied to structure the free text and extract relevant information from it [15].

The level or degree of structure can vary also for the structured data, for example, even if the type of a lab test is coded, the result of the test might be a in free text with different units used for the same test. Such information can be regarded as semi-structured and therefore, additional pre-processing is required also for these types of events.

### Data

The health records collected for this study comes from Karolinska University Hospital in Stockholm from the years 2007-2014 [16]. These records contain coded information such as diagnosis codes, drug codes, and procedural codes; semi-structured information such as lab test results; and free text. From this data set, the 1723 patients with an ICD-10 diagnosis code representing cervical cancer, i.e. a code starting with C53, were selected as the case group. Next, a control group was created where each patient in the C53 case group was matched on age to ten other control women, who did not have any C53 diagnosis on record in the data.

Since one aim of this work was to investigate the events leading up to a first diagnosis of cervical cancer, only cases with EHR data before the diagnosis date of cancer were included, and all patients with the code Z854C (previous cervical cancer) were also filtered out, leaving 1,321 cases and 16,212 controls. Five basic event types were extracted from the ERHs for both cases and controls: clinical entities found in the free text notes, diagnosis codes, drug codes, lab results, and procedure codes. The extracted data were then divided into intervals, for the first of which, all data up to the day before diagnosis were included. For the next interval, only data registered at least 14 days before diagnosis were included and so on in intervals of 30 days up to a year before diagnosis. The available data was divided into two parts, a development set containing 20% of the data and a test set with the remaining 80%. The development set was set aside for hyperparameter tuning of the classifiers and for feature selection. The test set was used to evaluate the selected classifiers through a process of 10-fold cross-validation, whereby ten rounds of training and testing was performed with 90% of the data used for training and 10% for testing for each round. The development set was not included in the final evaluation of the classifiers.

### ICD codes

**Fig 1:**
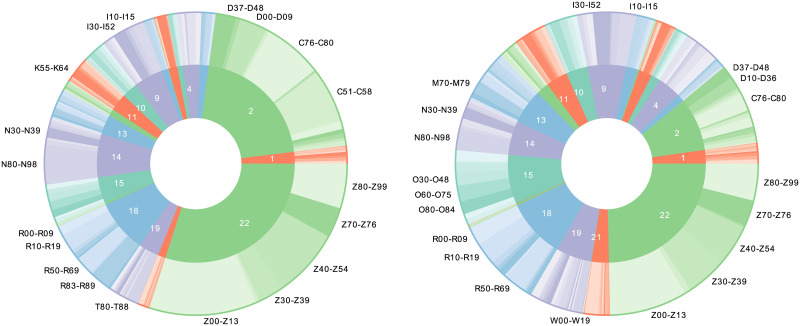
ICD chapters and sections for the case group and the control group. The left part corresponds to the case group and the right part the control group. The inner circle represents the ICD chapters and the outer circle the ICD sections included in the chapter; the labels show the sections with the most frequent codes.

The health records contain diagnosis codes from the Swedish version of the ICD-10 codes (International Statistical Classification of Diseases and Related Health Problems—Tenth Revision). ICD-10 codes are hierarchically arranged with 22 chapters at the highest level, these chapters are further divided into sections, subsections and full codes. This makes it possible to include both very fine-grained information in form of the full diagnosis codes, as well as higher level information, such as chapters and subsections. Table 1 gives an example of the ICD-10 hierarchy for the code C53.0. Fig 1 provides a visualization of the distribution of ICD-10 codes for the control group and the C53 case group. Comparing cases and controls, it can be noted that many of the patients in the case groups have experienced other types of cancers; 38% of cases and 22% of controls had a diagnosis code from chapter II of ICD-10, which is the chapter containing codes for tumors/neoplasms. Additionally, a large part of these codes came from the section representing codes for malignant neoplasms of the female genital organs, C51-C58, other than cervical cancer.

**Table 1:** Example from the ICD hierarchy.

Level	Example	Size	In data
Chapter	C00-D48, Neoplasms	22	22
Section	C51-C58, Malignant neoplasms of female genital organs	264	252
Subsection	C53, Malignant neoplasm of cervix uteri	2049	1409
Code	C53.0, Malignant neoplasm, endocervix	33564	5368

The top of the hierarchy consists of 22 different chapters, where chapter II comprises codes for neoplasms. Further down in the hierarchy, more detailed information is included in the codes. The column titled Size gives the number of different codes for each level in the complete code hierarchy and the last column (In data) shows the number of different codes included in the current data sets.

### ATC codes

The next feature type are the ATC codes from the Anatomical Therapeutic Chemical Classification System representing drugs. As with ICD-codes, these codes are hierarchical and therefore it was possible to represent them with different levels of detail. Table 2 shows an example of the included ATC levels.

**Table 2:** Example from the ATC code hierarchy.

Level	Example	In data
Main group	N, Nervous system	17
3rd level subgroup	N02B, Other analgesics and antipyretics	219
4th level subgroup	N02BE, Anilides	503
Full code	N02BE01, Paracetamol	1063

The last column of the table shows how many different codes appear in the current data set for each code level.

### Clinical entities extracted from text

Most of the data extracted from the EHRs were in the form of free text notes, and different approaches have previously been used to create representations of the free text in EHRs. One possibility is to map the text to some existing ontology or terminology. Roque et al. [17] matched free text to ICD codes to enrich coded information extracted from EHRs. This has the advantage of getting a coded representation, practical for interpretation and machine learning purposes, however, mapping directly from clinical text to standard terminologies can lead to low recall [18], as the language used in clinical text differs from the standardised language in terminologies. Tools for matching text to terminologies, such as MetaMap [19], are not available for Swedish. Another approach, which also reduces the size of the feature space, was applied by Miotto et al. [12]. They included free text in their model by extracting entities from clinical notes using the Open Biomedical Annotator and topic modeling, this approach was effective for predicting diagnoses, but has the drawback of reduced interpretability.

**Fig 2:**
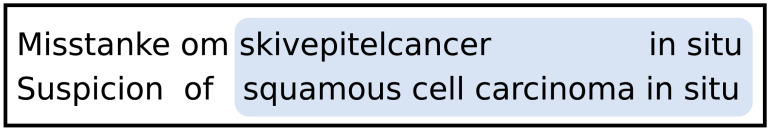
Example of patient record text. This example contains one multiword Disorder “squamous cell carcinoma in situ”.

Here, named entity recognition (NER) was applied to the free text notes belonging to cases and controls. Using NER allowed for extracting the most relevant parts of the text, and compared to including the full texts in a bag-of-words model, named entities makes it possible to represent multiword expressions such as *diabetes typ 2* (diabetes type 2), and *cancer in situ*. To extract these entities from the free text, a bidirectional long short-term memory (bi-LSTM) network was trained on notes annotated by two medical experts. The notes used as training data for the network were annotated for the entity types Body part (2103 annotations), Disorder (1059 annotations) and Finding (4501) annotations. For this work, findings and disorders were included as events and following the SNOMED definitions, a “Finding” represent both normal and abnormal observations regarding a patient while a “Disorder” always is the result of an underlying pathological process, see Fig 2.

A large corpus of clinical texts was used to generate word2vec [20] embeddings of the texts as input representation for the network. This corpus contained 1.2 GB of text from Swedish EHRs with a complete vocabulary of about 300,000 words. The properties of the LSTM network are described in detail in Weegar et al. [21]. This network was applied to all clinical notes for the cases and the controls to extract all mentions of findings and disorders from these texts which contained on average 2300 tokens for each patient. Next, negation detection was used to identify negated entities using a rule-based module, NegEx, adapted specifically to the domain of Swedish clinical text Swedish [22]. This was an important step because clinical notes often contain documentation of discussions and reasoning around a potential condition in text and it is thus particularly common that findings are negated. Indeed, in this material up to ten percent of findings and six percent of the extracted disorders were actually in the negated form. Similarly, to mitigate the risk of including information regarding individuals other than the actual patient to whom the record belonged (deriving from e.g. discussions on family history of disease), clinical notes with headings related to family members were excluded. The use of NER for event extraction resulted in 148 events for the controls on average. For the cases, only events occurring before the C53 diagnosis were included, on average 102 events.

The extracted events were lemmatized which reduced the scarcity of the free text data as lemmatization maps inflected versions of a word to the same basic representation. By using lemmatization words such as hudirritation/skin irritation (singular) and hudirritationer/skin irritations (plural) will be considered as the same event. Similarly, the event hostade/coughed (past tense) will be joined with hostar/coughs (present tense). But even after lemmatization, the number of different events extracted from the texts is very large. There were about 75,000 different events appearing at least two times. This is partially due to the possibility of describing the same event in many different ways in writing, for example, “fractured patella” and “patella fracture” refers to the same type of event, but will be considered as two different events as their surface forms are different. Therefore, to further reduce the feature space, two additional preprocessing steps were applied to the textual events. Firstly, a spelling normalization module was used to group different spelling variations of the same concept. This grouping was achieved through calculating the edit distance between each pair of events. A Levenshtein edit distance of two strings is a measurement of how different the two strings are, and is calculated by counting the number of insertions, deletions and substitutions of characters that is required to make two strings equal [23]. Groups of events were formed where each member in the group had at most an edit distance of one from some other member of the group and the same first letter as all other members in the group, as it was found that allowing larger distances introduced errors. For multi-word expressions the distance was calculated per word, and it was also required that each included word had the same first letter. The spelling differences in the text data were mainly the result of misspellings, but also of inflections or instances of writing the same concept as a either a compound word or a as two separate words. After grouping the events using edit distance, all variations were exchanged with the most frequent surface form. One example of such a group is the spelling variations for thyroid cancer:

{thyreoideacancer: tyreoideacancer, thyroidecancer, thyreoidacancer, thyreoidcancer, tyroideacancer, tyreoidecancer, thyroidea cancer, thyroideacancer, thyreoidecancer}

where each variation was mapped to the most frequently occurring form “thyreoideacancer”.

This normalization mapped about 49,000 different surface forms of events into about 18,000 different groups.

Next, a hierarchical representation of the events extracted from the free texts was created. The hierarchy was constructed by first sorting the events extracted from the texts according to their length and placing one-word entities at the top of the hierarchy. Next, for each level, any event that contained all words of an event on the higher level was added as a child node of that event. In this hierarchy the one-word event “leukaemia” at the top level had the child nodes “acute leukaemia” and “chronic leukaemia”, where “chronic leukaemia” in turn had the child nodes “chronic myeloid leukaemia” and “chronic lymphocytic leukaemia”. Events further down in the hierarchy became gradually more detailed as they often contained some type of modifier to its parent node such as “normal”, “serious” or “malignant”, capturing many relevant relationships between events.

A hierarchy of four levels was created with one-word entities at level one, and events containing four or more words at the fourth level. It is worth noting that when representing the textual events for a single patient, the longer, low-level events had influence over higher level representations in the same way as for the ICD or ATC code hierarchies. Using the ICD code hierarchy, a diagnosis can be represented by the corresponding full low-level code or by the section or chapter it belongs to. In a similar way, the lower level event “chronic myeloid leukaemia” can be represented by the higher level event “leukaemia”.

This hierarchy of textual events was however different from the ICD and ATC code hierarchies in two ways. Firstly, each child node could have more than one parent node: an “infected wound” had the parent “wound” and the parent “infected”. The second difference was that while the top levels are the smallest in the code hierarchies, the opposite is true for the textual events. There was a higher number of short textual events, and the short events also make up a larger part of the total set of textual events. The top level of the hierarchy contained 25,587 different events, followed by 29,665 events at level two, 8,254 events at level three and 3,586 events at level four.

### Lab results

The included data for lab results consists of the type of test and the result of the test. Including the value of test result can be too fine-grained for the task of prediction, since many tests are rare [24]. Therefore, the result is represented as being either inside or outside the reference range for a test value. For example, the result of a Hemoglobin test can either be in the normal range for Hemoglobin, above the range or below it. Therefore, each lab result in the included health records will be represented by one of three different features depending on the outcome of the test.

### Procedure codes

Procedure codes classify procedures including surgical procedures, medical investigations, preventive measures, and treatments, for example, the code AK044 corresponding to an ultrasound of the kidneys. The codes are used in health records for statistical and administrative purposes, and have been included as an event type in this work.

**Fig 3:**
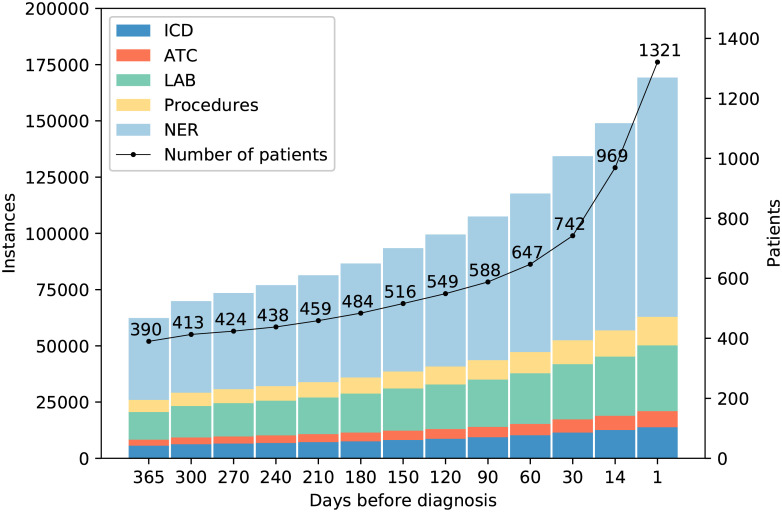
Available data for the case group. The x-axis denotes the number of days before a diagnosis, and events only appearing one time in the data set have been excluded. At 90 days before a diagnosis 107,468 events were available for 588 patients.

A number of the events that were extracted from the EHRs were unique, meaning that they only occurred for a single patient. Since such events cannot contribute to the diagnosis prediction, but only increase the scarcity of the data, they were removed at this stage, leaving in total 87,219 different events in the data set. Each case had on average 94 different events and 187 events in total before the diagnosis. For the controls, the corresponding numbers were on average 136 different events and an average total of 282. The reason for the larger number of events for the control group is that only data up to a cervical cancer diagnosis is included for the case group and all events after the diagnosis were discarded. For the controls on the other hand, all documented events were kept even if they occurred after the time of their matched case’s diagnosis. Since the objective of our study was correct classification based on EHR events with potentially low expected contrast between cases and controls, we opted for this choice to 1) maximize the control information for the classifiers to be trained on, 2) minimize the risk of misclassification of disease status by ensuring to the best of our knowledge that no hospital-based female controls were diagnosed with cervical cancer later during the study period and 3) to increase generalizability to a real-life clinical situation where EHRs are available but disease status of the individuals is not already known. Fig 3 gives an overview of the available data for the different time intervals. However, some events will appear outside of the study period, leading to data censoring [4, 5]. Important and informative events could have occurred before the start of data collection and this also means, as in most case-control or other studies, that we do not know whether some members of the control group develop cervical cancer *after* the study end point, when no more EHR data was available to us. However, this is a well-known fact resulting from this type of study design and does not invalidate the comparison made during the actual study period defined.

### Classification experiments

Four different classifiers were used to evaluate the appropriateness of the different feature types for classification of future C53 cases. These classifiers were Random Forest, Complement Naive Bayes, Bernoulli Naive Bayes, and Support Vector Machines, all implemented in Scikit-learn [25]. The first classifier, Random Forest, is an ensemble classifier robust to noise and large feature spaces [26], Complement Naive Bayes [27] is capable in cases of data sets with class imbalance, Bernoulli Naive Bayes additionally takes absence of features into account for classification [28], and Support Vector Machines (SVM) is a classifier well suited for high dimensional spaces [29].

This classification task can be understood as a text classification problem, where each event extracted from a patient record corresponds to a word and each patient is represented by a vector with the same dimension as the complete vocabulary of events. For Random Forest and Complement Naive Bayes, the input vectors consisted of the raw counts of events. For Bernoulli Naive Bayes, binary input vectors were used, and for the SVM classifier, normalized vector counts were used as input. Binary vectors correspond to if a patient ever experienced an event, and count vectors also represent how many times each event occurred. Another possibility is to use tf-idf (term frequency-inverse document frequency) weights. The idea behind tf-idf is to give more weight to the events that are representative for individual patients. However, using tf-idf did not improve classification results.

## Results

### Event types

**Fig 4:**
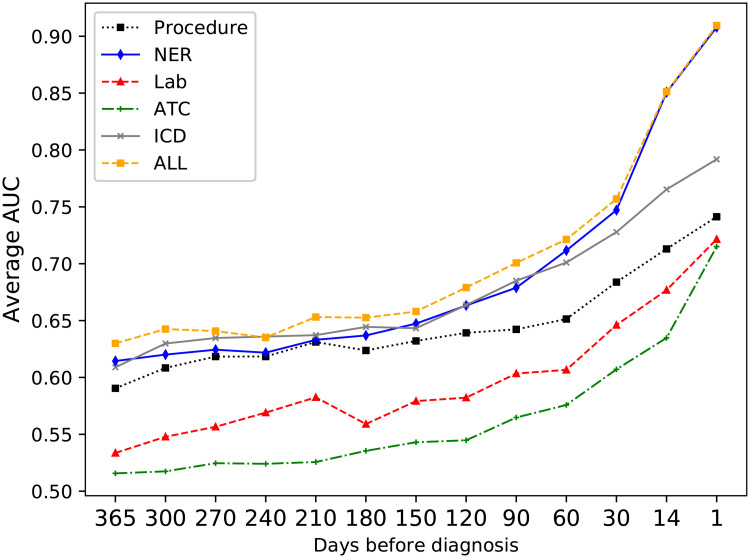
Average AUC for the different event types over time.

Each type of event was evaluated individually for its ability to correctly classify the patients. Fig 4 shows the average AUC using the four classifiers over time for each event type (ICD codes, ATC codes, Procedure codes, Clinical entities, and Lab results). The average AUC was calculated as the sum of the scores *AUC*_*i*_ for the individual classifiers, divided by the number of classifiers: $AUC_{avg}=\frac{1}{4}\left(AUC_{1}+AUC_{2}+AUC_{3}+AUC_{4}\right)$. The most informative event type for the classifiers were the clinical entities extracted from the text, as the highest AUC scores were achieved using only the text entities.

### Event levels

Since the data in EHRs typically are noisy, high dimensional and sparse, it is necessary to investigate data representation, how the information included in the records should be presented to the model [4]. In this work, both which types of feature to include and also the detail with which those features should be represented was evaluated.

**Fig 5:**
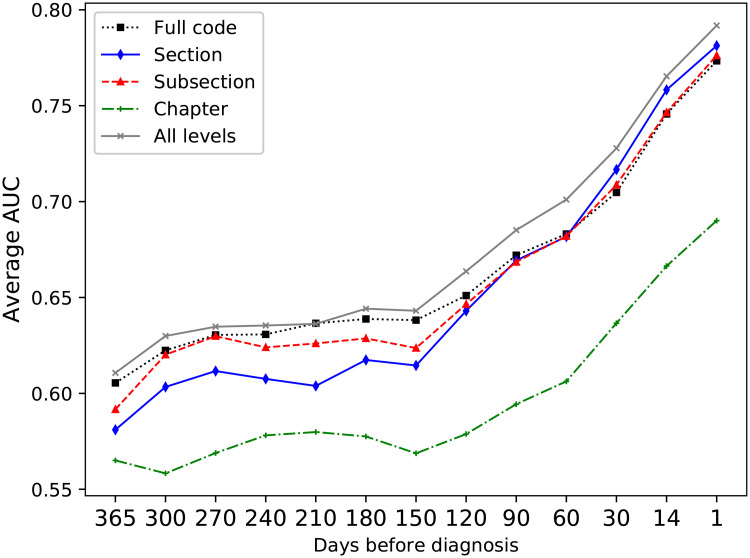
Average AUC for the ICD levels. Comparison of the average AUC over all four classifiers for the different ICD levels. Combining all levels gives a better performance than any individual level.

**Fig 6:**
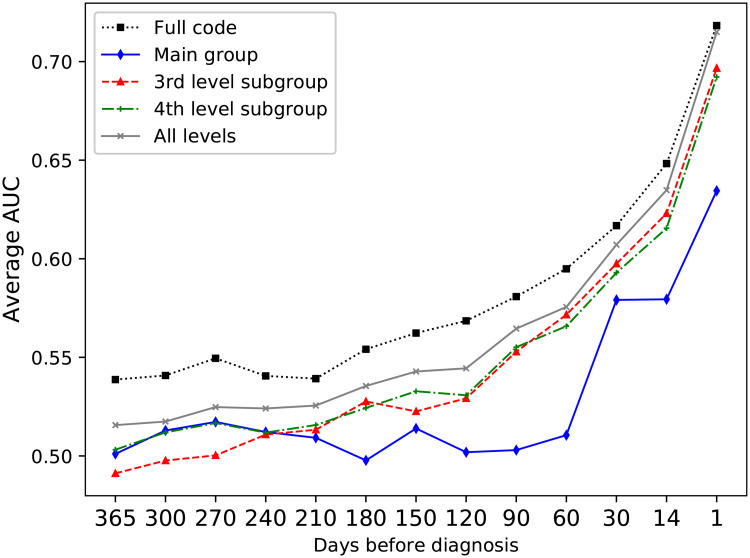
Comparing average AUC over all four classifiers for the different ATC levels. The y-axis represents average AUC over the classifiers. Here, using the full ATC codes gave the highest performance.

It has previously been found that including features derived from several levels of hierarchical clinical codes improves the performance for predicting adverse drug events from EHR data [30], and as the input events can be represented with different levels of detail, the next set of experiments aimed to evaluate the most suitable representation of the events in this regard. Using the most detailed levels, such as a full ICD-10 code, gives very detailed information, but a very sparse data set. By including the higher-level representations, the sparsity of the data can be reduced. Fig 5 shows the average AUC over the four classifiers for the different ICD-10 code levels on the development data set. The best results were achieved when including all levels in the ICD hierarchy. For the different ATC code levels, Fig 6), it was however found that higher results were achieved only using full ATC codes.

**Fig 7:**
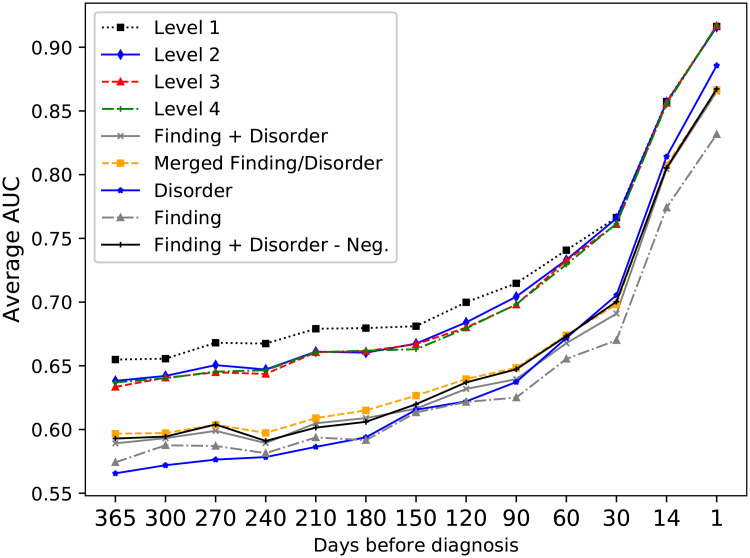
Classification results for the different representations of text events. Level 1-4 refers to the different levels of the textual event hierarchy. The y-axis represents average AUC over the four classifiers. Using Level 1 of the event hierarchy provided the highest scores.

For this study, a hierarchy of events was also created for representation of clinical events extracted from free text notes, and the suitability of this representation was compared to including each event in separate. Fig 7 gives an overview of these results. By first using the non-hierarchical representation, it was found that it was beneficial to merge the classes Finding and Disorder for most of the time intervals. One additional question was how to deal with negated events mentioned in the text as there are two possibilities, either to exclude them from classification or include them as a separate event type; here the difference in performance for the two approaches was very small and they were therefore excluded for the remaining experiments. For the hierarchical representation, it was possible to include the full hierarchy or only parts of it. Including the highest level outperformed including lower levels, but all hierarchical representations gave better prediction performance compared to the best non-hierarchical representation and provided a reduced feature space.

### Feature ranking

The purpose of feature selection is to improve the performance of a classifier by only including a subset of the features and excluding irrelevant or redundant features [31]. One method for feature selection is to rank the individual features according to their relationship to the class label, where features with the highest ranks are considered to most impactful [31]. The ranking can be found using different statistical tests or measures such as mutual information [32]. After the ranking is found, the features are sorted according to their rank and features below some threshold can be considered as uninformative and therefore discarded [32].

**Fig 8:**
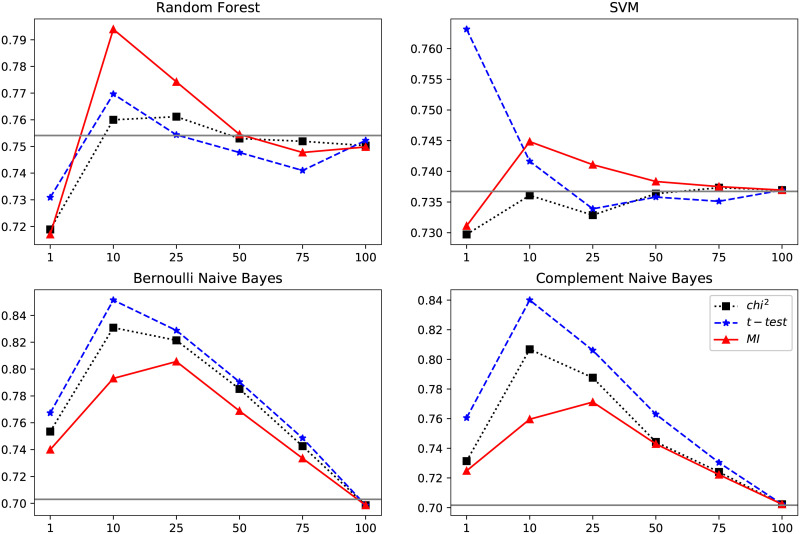
Average AUC scores over the four classifiers using ranked events. The events were ranked using t-test statistic (t-test), mutual information (MI) and chi-squared testing (ch*i*^2^). The horizontal line shows the performance when including all features (i.e. without ranking). 100 means including all informative events, i.e. events with a rank above zero, 50 means including 50% of all features with a rank above zero. Including the top 10% of all events was most effective for all classifiers except SVM for which using the top 1% was the most effective choice.

Here, the features were individually ranked to identify the features most related to the diagnosis and therefore also most important to include when training the classifiers. Three different feature ranking methods based on three different measures were evaluated: Mutual information, Chi-square test and the t-test statistic. Each of these measures provided a scoring for the association between the events and class membership (a future cervical cancer diagnosis). All events were ranked according to the three different measures and the classifiers were then trained using subsets of the events with the highest ranks. Fig 8 shows the impact of training the classifiers on the top ranked events compared to including all available events in the training.

Overall, ranking the events using the t-test [33] gave the most improvement in terms of classification results. This test gives a high score for a feature which 1) has a low variance within the group (i.e. within the case group or the control group, respectively) and 2) different mean values between the case and control groups, meaning that an event that is common in both groups received a low rank, while an event that was frequent for many members in case group while uncommon among the controls received a high rank. The highest ranked features are listed in Table 3. Recursive feature elimination, where the features contributing the least to classification are iteratively removed from the model was also evaluated, but did not improve results.

**Table 3:** The 25 events with the highest ranks according to the t-test.

Event	Event type	Description	% cases	% controls
cervixcancer	Text Entity	cervical cancer	43.603	0.345
cervixca	Text Entity	cervix ca	7.949	0.037
mikroinvasiv	Text Entity	microinvasive	9.765	0.037
skivepitelca	Text Entity	squamous cell ca	6.662	0.173
cin 3	Text Entity	cervical dysplasia, grade 3	14.459	0.574
medelhög diff	Text Entity	moderately diff	12.339	0.962
skivepitelcancer	Text Entity	squamous cell carcinoma	31.643	0.956
invasivt växa	Text Entity	invasive growth	8.251	0.827
skivepitelcancer in situ	Text Entity	squamous cell carcinoma in situ	6.435	0.222
lågt diff	Text Entity	poorly diff	9.841	0.648
Z031	ICD full code	Observation for suspected malignant neoplasm	7.873	1.382
överväxt	Text Entity	overgrowth	6.510	1.024
D06	ICD subsection	Carcinoma in situ: Cervix	8.857	0.617
D069	ICD full code	Carcinoma in situ: Cervix, unspecified	8.478	0.592
lättblöda	Text Entity	easily bleeding	11.734	2.233
AV033	Procedure	Evaluation before surgery	12.339	3.855
LDC03	Procedure	Cervical conization using diathermy or laser	5.223	0.907
C51-C58	ICD section	Malignant neoplasms of female genital organs	15.443	2.054
XLE00	Procedure	Colposcopy	8.251	1.252
cellförändring	Text Entity	dysplasia	11.052	1.999
cancer in situ	Text Entity	cancer in situ	6.586	0.827
postmenopausal blödning	Text Entity	postmenopausal bleeding	6.283	0.746
LDA20	Procedure	Biopsy of portio	8.781	0.944
breddöka	Text Entity	increased width	6.510	0.981
låg diff	Text Entity	poorly diff	7.040	0.592
C54	ICD subsection	Malignant neoplasm of corpus uteri	8.100	1.147

Events associated with less than 5% of cases have been excluded. The description column gives translations of the Swedish textual entities and the words “ca” and “diff” are abbreviations of “cancer” and “differentiation”, respectively. Since lemmatization has been used, an entity such as mikroinvasiv (indefinite form) also represents mikroinvastivt (definite form) and mikroinvasiva (plural form).

### Classification results on the test set

**Fig 9:**
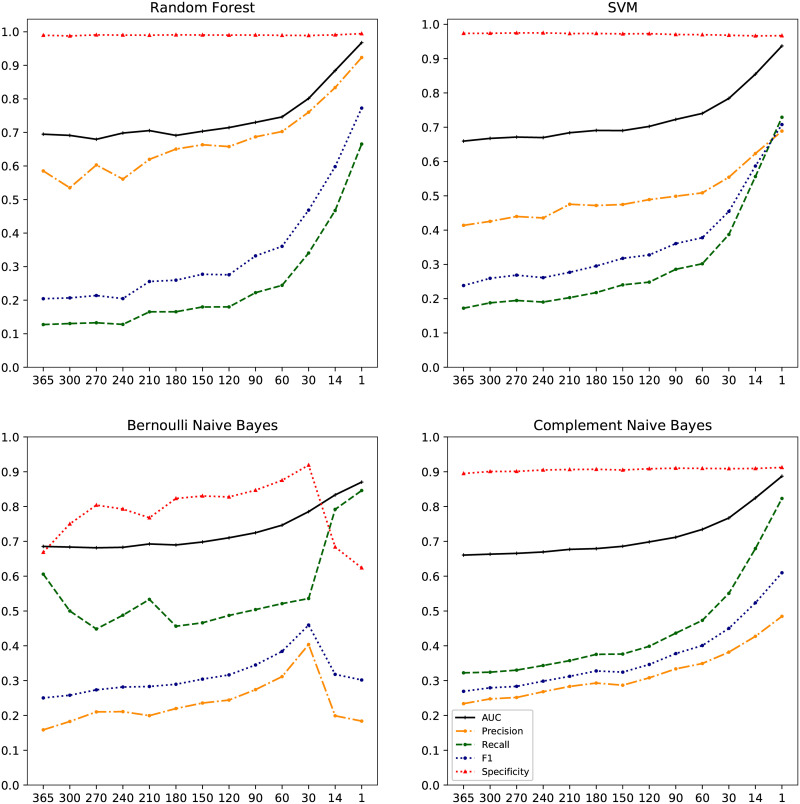
Prediction results. Precision, recall, F1-score, AUC and specificity over time per classifier for predicting cases of cervical cancer. The x-axis shows the number of days before diagnosis.

Finally, the four classifiers were trained and evaluated using precision, recall, F1-score and AUC (area under the ROC curve). These results were obtained using 10-fold cross validation on the test set with only the events given the highest ranks on the development data, see Fig 9. The performance of the different classifiers in terms of AUC was similar with the overall highest scores for the Random Forest classifier, ranging from 0.70 a year before diagnosis, up to 0.96 when including all data up to one day before diagnosis. Random Forest also achieved the highest score for precision (0.53-0.92) while Bernoulli Naive Bayes reached the highest recall scores ranging from 0.45 to 0.85. Sensitivity, i.e. the ability to identify the cases (true positives), was highest with the Bernoulli Naïve Bayes classifier, with a proportion of 0.61 (61%) of (future) cases correctly identified at 1 year before diagnosis. This metric ranged from 0.12-0.32 for the other classifiers. In absolute numbers, this translated to 190/314 (61%) cases identified for Bernoulli Naïve Bayes classifier, 101/314 (32%) for Complement Naïve Bayes, 54/314 (17%) for SVM and 40/314 (13%) for Random Forest. Specificity, i.e. the ability to identify the controls (true negatives), was high for all classifiers, particularly for Random Forest and SVM classifiers with a specificity over 0.97 for all time intervals. The specificity was slightly lower for the Bernoulli Naive Bayes classifier (between 0.62 and 0.92) and for the Complement Naive Bayes classifier (between 0.89 and 0.91).

## Discussion

There is currently great interest in investigating real-life applications of health care-derived patient data, and how to best use the wealth of data collected on a routine basis, for the improved health of all. One particularly challenging, yet important, way forward would be to track symptom development trajectories for patient groups over time, and then use this information to be able to predict disease earlier in future cohorts of patients, who may only have recently started on similar trajectories. Ideally, such an approach could yield significantly earlier diagnosis, with improved survival as a result.

In this context, our principal aim was to investigate how to best represent hospital-based EHRs for predicting future cases of cervical cancer through machine learning. The clinical entities extracted from the free text records gave the most accurate classification of future cases, and using a novel hierarchical representation of these events gave an improved predictive performance. But overall, using a combination of events was more effective compared to any single event type, see Fig 4.

The clinical named entities were also the event type able to predict the disease at the earliest time point. This is likely partially because the diagnosis is discussed in free text before the diagnosis code is set, and that there are mentions such as “suspected cervical cancer” appearing in the free text notes before the diagnosis code is given. Thus, this finding might represent a health care process-related artefact. Regarding the level of detail with which the events should be represented, we found it to be most effective to include several different code levels for the diagnosis codes, but full ATC codes outperformed the multilevel ATC representation.

An early prediction of the diagnosis would be beneficial, and in our study an AUC of 0.70 was achieved a full year before diagnosis increasing to 0.97 closer to the date of actual diagnosis. This result is perhaps expected, as it is known that it more challenging to predict events occurring further away in time [5]. The lower early prediction results were also likely impacted by the fact that there were relatively fewer cases with over a year of documented medical history, as it is naturally more difficult to create an accurate model from a smaller data set. There simply was not enough data for our classifiers to learn from, that differed between cases and controls. Like any screening test for detection of prevalent or incident disease, our approach relies on finding clear contrasts in the test metrics under investigation between current/future cases and controls. In this study, we could not identify any very strong patterns of previous care or test results that were strongly associated with only being a future case, one year later. A high proportion of later case women simply did not have such characteristics in a combined assessment of their health records from the Karolinska University Hospital database. This could be due to a) an absence of relevant predictors at this time point, b) an inability for the current study to find such predictors, or c) a combination of a and b. This is a large motivation for why we will endeavour to obtain primary care data for the next phase of research, to address point b) above and improve the granularity of EHR data in the prediction of disease. We could then investigate whether addition of more data will lead to improved classification and at greater distance ahead of diagnosis.

As a result of the so far limited data density in our study, the recall/sensitivity at one year before diagnosis was modest. The classifier with the highest recall—the Bernoulli Naive Bayes—correctly identified a modest level of 61% of the cases at this time point. Almost 40% of cases were thus incorrectly classified as being without (future) disease. That said, we would propose that given the invasive nature of surgical and radiation treatment to the cervix, any earlier detection (and potentially less invasive treatment needed) of the cancer would be worthwhile, even if not all cases were discovered a full year before. In this context, our classifier’s sensitivity could be considered acceptable, so long as the specificity at the same time point was very high. However, since that was not the case with the Bernoulli Naïve Bayes, we conclude that further development work is needed to obtain a reliable prediction and classifier performance.

Finally, we note that individual feature importance, as determined by a machine learning model, have previously been used to evaluate risk prediction models and the relationship between events and outcomes for colorectal cancer [11], but there is no method for directly determining which findings mined from health data actually represent novel knowledge [4]. In this study, high rankings were given to individual events that are known to sometimes precede a cervical cancer diagnosis—this would include items such as mentions of cancer in situ (also cervical intraepithelial neoplasia grade 3 and “dysplasia”), the procedure code representing “Biopsy of portio”, and the ICD-10 code D06 (Unspecified location of cervical cancer in situ), these findings indicate that the models learned from relevant non-spurious information.

## Conclusion

The results of this study shows the potential significance of including free text in prediction models based on health records, as the events described in free text were found to be the most important component among the features included for the diagnosis prediction.

For the current specified clinical application, we observed only modest discrimination performance at one year before diagnosis. Thus, for our future work, we would like to extend the model here to primary care data as well. An increased frequency of visits to primary care has been observed prior to a subsequent cancer diagnosis [34], and including primary care health records would provide longer patient histories entailing more information to learn from.

Furthermore, the order of the individual events was not considered, only the distance from an event to a diagnosis. For future work, it should therefore also be of value to apply a sequential model such a recursive neural network to this data set. Finally, it should be noted that we here chose cervical cancer as a starting point, due to the severity of the disease and a well-defined disease development process which allowed us to continuously evaluate reasonability of results. However, this disease is relatively rare and even though we had access to a large dataset, the number of events was such that we only reached moderate prediction capabilities a year before diagnosis. Still, we propose that the same approach delineated here could be applied for prediction of other disease entities as well, and it should be of significant informatic and clinical interest to observe which predictions may result, especially if the disease is relatively common and the data density greater.
